# Gender-based violence programming in times of COVID-19: Challenges, strategies and recommendations

**DOI:** 10.3389/fgwh.2022.952688

**Published:** 2022-12-06

**Authors:** Mridu Markan, Radhika Dhingra, Mehak Segan, Vandana Dabla, Moni Sagar, Sharmila Neogi, Shrija Dey, Nayan Chakravarty

**Affiliations:** ^1^ USAID's Momentum Country and Global Leadership: India-Yash, Jhpiego, New Delhi, India; ^2^ USAID, New Delhi, India

**Keywords:** gender based violence, COVID-19, GBV programming, GBV India, GBV challenges

## Abstract

Gender-Based Violence (GBV) remains the most challenging and threatening manifestation of gender inequality in Indian society. The outbreak of COVID-19 in India increased the risk of exposure to GBV, often compared to the “shadow pandemic". Girls suffered disproportionally compared to boys during the pandemic -from being pulled out of schools, facing movement restrictions, and being more susceptible to forced marriage and household violence. Pre-existing gender inequalities and regressive gender norms, along with economic instability, also contributed to creating a milieu for violence to thrive. Additionally, the pandemic also challenged GBV service provision and program implementation at the community level. To meet the increasing needs of women and girls during the crisis, national and local civil society organizations attempted to adapt GBV programming and promote innovative approaches to tackle GBV. The secondary review provides insight on the GBV impact due to the COVID-19 pandemic and provides an overview of various challenges at the level of individual, community, institution, and policy. The literature review also highlights strategies adopted to combat GBV in private, public and cyberspace.

## Introduction

Gender-Based Violence (GBV) is directed at an individual or group based on actual or perceived biological sex, gender identity, gender expression, or perceived adherence to socially defined norms of masculinity and femininity (1). Worldwide 35 percent of women have endured violence from an intimate relationship or non-partner sexual violence. Intimate partner violence is more widespread in low- and middle-income countries such as Africa and Southeast Asian countries. In contrast, non-partner sexual violence is most common in high-income ones. Globally, the intimate partner is responsible for 38 percent of all women killed (2). According to the National Family Health Survey (NFHS) 5, in India, 44.5% of married women have experienced spousal violence at least once in their lifetime. Generally, GBV differs across and within different societies depending on the individual, the family, the community, and the broader national context. GBV is known to be widespread in all settings; nevertheless, emergencies undermine established protective systems and produce a variety of scenarios that can lead to various forms of violence, abuse, and exploitation (3, 4).

## Impact of COVID-19 pandemic on gender-based violence

The COVID-19 pandemic resulted in a shadow pandemic of violence, i.e., an exponential global increase in GBV. In India, apart from the surfacing of economic and social stress caused by the SARC-CoV-2, it also revealed deep-rooted patriarchy, gender inequalities, and preexisting harmful social norms fueling GBV. The social isolation measures and movement restrictions imposed to prevent the further spread of the pandemic made women and girls more susceptible to violence at the hands of family members, partners, and those living within their homes. Various studies have demonstrated that most GBV cases in emergency conditions are perpetrated by a known individual (5–9). Comparative evidence from the NFHS 4 and NFHS 5 illustrates that young women (18 years–29 years), who have experienced sexual violence by the age of 18 years, have risen from 10.3% to 11.0%, indicating an increase in domestic and sexual violence during Covid-19 pandemic-induced lockdowns. In addition, it was further established that more efforts are required to assess the actual impact of COVID-19 on gender indicators across India (10). Women and girls who experienced violence at home were trapped with their perpetrators during the COVID-19-induced lockdown. There was a 131% increase in domestic violence complaints in May 2020 in districts that saw the strictest lockdown measures relative to districts that saw the least stringent measures (11).

The series of lockdowns across the country pushed women from being cut from access to services and information, worsening women's social and economic situation (12). The primary responsibility of unpaid household work fell on women disrupting work-life balance (13). Statistics reflect that a woman spent up to 353 min daily on household work, as opposed to 52 min spent by men. An increase in gender-based household maltreatment was also observed wherein women were subjected to violence by husbands, brothers, fathers, fathers-in-law, etc. (14). In the year 2021, National Commission for Women (NCW) reported a 30% increase in the number of complaints received than the year 2020.

The maximum of these complaints (36%) was under the right to live with dignity clause, also considered emotional abuse. The pandemic not only had a detrimental impact on the physical and mental health of women and adolescents but also led to an increase in child marriage. From May–July 2020, there was a 33% increase in reports of child marriage to India's first government-operated 24-hour toll-free phone outreach for children (15). The pandemic also increased instances of sexual exploitation wherein landlords were exploiting women physically under the pretext of offering them cheaper accommodation (16). During the lockdown, domestic violence cases continued to increase alarmingly, making survivors vulnerable to violence, for they could not leave spaces of violence or even access health care (17). The abusers were able to impose fear and violence due to isolation, leading to increased marital violence (16). COVID-19 made access to sexual and reproductive health services difficult for women to connect with health care providers, thereby increasing the risks of unprotected sex, pregnancy, forced sex, and marital rape. Loss of jobs because of the pandemic increased financial insecurity for both men and women. Research has demonstrated that unemployed males tend to exert more control over their partners, making them vulnerable to violence (17). The pandemic disempowered women irrespective of their caste and class. The domestic space of the household continues to be a space to exert patriarchy, power, and dominance and is indicated by women and the continuation of patriarchy through marriage and child-rearing (18).

## Gender-based violence programming in times of COVID-19

Gender-specific programming in any area refers to unique programme models and services that address the unique needs of a specific gender group (19). Due to unequal power-sharing in our society, young girls and women are most vulnerable to gender-specific dangers (20). As the pandemic increased the risk of exposure to gender-based violence (GBV) and challenged service provision and program implementation, GBV programming remains even more essential and life-saving. Gender-based Violence Programming ensures a comprehensive response to GBV, projects seeking to increase the protection of survivors, prosecution of perpetrators, and prevention of future crimes. It also emphasises that GBV programs are comprehensive, integrated, sustained over time and interlinked for holistic strengthened response.

During the pandemic, the health systems were overwhelmed, leaving women with limited access to the prevention of GBV services. The focus shifted to providing essential services and preventing the spread of COVID-19 (21–23). The pandemic also challenged GBV service provision and program implementation at the community level as many organizations prioritized providing first-line COVID-19 relief services. The response to COVID-19 in India did not consider the widened gender, social and economic inequality present. The lack of awareness and absence of knowledge on how to report GBV contributed to further deterioration of the condition of women who were trapped in their own homes. To meet the increasing needs of women and girls during the crisis, national and local civil society organizations attempted to adapt GBV programming and promote innovative approaches to address it (24).

The public and private sectors introduced various hotlines, but many individuals could not notify the authorities *via* hotlines for they did not know they existed. It was challenging to ensure the penetration and dissemination of these hotlines in rural areas (25). The pandemic revealed the gender disparity (26) worldwide, and organizations working on supporting women and girls saw their cuts when their services were most needed. Two hundred women organizations across thirty-eight countries saw budget cuts and forced attrition and reported limited access to the decision-making process. In India, efforts of the United Nations could only be seen through supporting existing interventions on GBV and suggesting innovative strategies to curb GBV. There is a dire requirement to increase government investment in prevention and include that as a part of minimum care (27). India's primary health care is dependent on the 1 million Accredited Social Health Activists (ASHA), 0.9 million Auxiliary Nurse Midwives (ANM) 17, and 1.4 million nutrition workers called Anganwadi workers (28). During the heightened pandemic, these frontline workers, who majorly were women, reported increased physical violence while trying to prevent the spread of infections in their villages (29). The prolonged negative impact of COVID-19 affects women and children and has significant cost implications on the country's overall development. With this in mind, the current study looks at some of the challenges faced by organisations in India to combat GBV during the pandemic, highlighting some of the innovative approaches adopted and deep-dives into some of the solutions and recommendations that can be adopted to prevent future shadow pandemics.

**Objectives of review:**

a. To gather insights on challenges faced by organizations working across India on GBV programming and strategies adopted to ensure undisrupted support to survivors of violence or those at extreme risk of violence during the COVID-19 pandemic.
b. To suggest actionable recommendations for researchers, policymakers, donors, and humanitarian organizations to steer GBV programming effectively in the context of the pandemic.

## Methodology

The review draws on peer-reviewed primary and secondary studies published in academic journals, summary meta-analyses, systematic reviews, other published reports, development program assessments and evaluations, and other relevant literature identified through systematic keyword searches and forward and backwards snowballing of key sources conducted across the internet using Google, Pubmed and Google Scholar. Both quantitative and qualitative studies are included in this review. The review was commenced from December- March'22. Within these searches, a keyword search strategy was adopted each thematic area, such as gender-based violence, intimate partner violence, domestic violence, spousal abuse, physical abuse, GBV programming, and challenges of GBV in COVID-19, India. Additional sources were retrieved based on the references in the publications identified in the initial search. The search was limited to English language sources.

## Challenges for gender-based programming in times of COVID-19 pandemic

**Figure 1:**
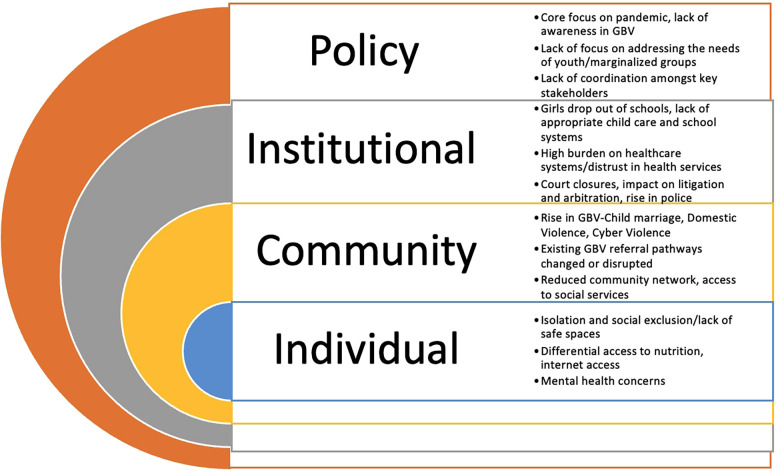
Key chellenges faced by civil society organisations while dispensing GBV services during the pandemic. This model is based on the Social Ecological Model (SEM) and is adapted in the context of GBV programming during the COVID-19 pandemic.

While the necessity to address GBV in the COVID-19 pandemic is undeniable, governments, social service providers, and other stakeholders' capacity to respond is far more problematic. Available funding for GBV programming is also limited in crisis settings, and decision-makers do not consistently prioritize GBV as a crucial component of preparedness and response operations. Health systems and national social services in LMIC nations afflicted by COVID-19 have become severely overstressed, with available resources frequently diverted to responding to COVID-19 patients, leaving fewer resources open for effectively addressing GBV (30). Key challenges elicited can be adapted to the social-ecological framework to reflect the individual, community, institutional, and policy challenges “Figure 1”.

### Individual challenges

The COVID-19 catastrophe has had and will continue to significantly impact the health and well-being of young girls and women (31). Girls and women encounter compounding burdens as they are over-represented in health-care systems, perform most of the unpaid care work in Indian households, face high risks of economic instability, and experience more significant threats of violence, exploitation, abuse, or harassment during times of crisis and quarantine (32). Female social exclusion begins early in life (33), and when combined with the national lockdowns and other movement restrictions, homes can become potential GBV hot spots. When the causes of violence become increasingly prevalent, survivors and those in danger in the absence of any safe places are more restricted than ever in their capacity to seek protection or other types of critical support (9). The pandemic has threatened India's precarious food security environment with a disproportionate burden on health and food problems. Women and young girls continue to face the consequences of increased unemployment, food supply disruptions, and a decline in international trade. This also has a direct bearing on pregnant women's food and nutrition consumption given food insecurity risks have been greater for women in COVID-19 crisis. There has been a reduction in financial security risks increasing gaps in the intra-household distribution of resources biased towards men and boys, which could further negatively impact women in the household (34). The existing gender digital divide (35) leads to not having access to phones, computers, or internet services, or being able to use them safely at home for communication, makes them even more excluded as they have to depend upon their husbands, fathers, or brothers for using the mobile phones and internet services (23). Additionally, those with access to phones and internet services, experienced technology-based violence during the pandemic.

### Community challenges

The COVID-19 pandemic has interrupted many current services, exacerbated pre-existing inequities, and worsened harmful effects on women and girls (36). Existing referral mechanisms and approaches to reacting to reported cases of GBV are likely to change or be interrupted in areas impacted by COVID-19. Depending on the setting, health facilities prioritising COVID-19 response may be less accessible as a point of access to essential services for GBV survivors, compelling them to either not report or narrate their cases to providers from other sectors who may not be appropriately qualified to respond to their disclosures (9). Moreover, women and girls at this time may not be able to reach their community networks. These organizations support and recognize abuse like local women's rights networks, teachers, faith leaders, and community development officers, which further deepens the issue (37).

### Institutional challenges

The pandemic has increased poverty levels and hunger and decreased access to education, and the risk of girls becoming child brides is also increasing. Child marriage is increasing in the country due to school closures during the lockdown, leading to huge human and economic crises (38). According to a recent UN Women survey, more girls than boys were left out of school during the pandemic, and 65% of parents surveyed were hesitant to continue girls’ education, turning to child marriages to save money. This can result in an entire generation of young women who lack access to education and employment possibilities (39). Several female students are facing the maximum brunt of the pandemic as their confinement within households in addition to their family's deteriorating financial conditions are creating increasing pressures on them to leave higher education to get married or take up jobs. Their plights are further compounded by increased pressure to do household chores and subjection to domestic abuses (40). Further, by 2021, the pandemic would have pushed 96 million people into severe poverty; 47 million will be women and girls, raising the total number of women and girls living on USD 1.90 or less to 435 million globally (41). Increased GBV-risks due to secondary impacts of COVID-19 also lead to higher child- and forced-marriage rates and related early pregnancies, which results in increased maternal and neonatal mortality. Globally, 10 million additional girls are at risk of child marriage due to COVID-19.

### Policy challenges

With the onset of the pandemic, the primary focus was on the prevention and treatment of COVID-19. Since GBV services were not recognised as essential health services during the start of the lockdown, services needed by the young girls and women were deprioritized, including shelters, health care services, police, and justice sector services. Around the world, shelters for survivors of gender-based violence have been closed or changed into homeless shelters, and reports of emergency hotlines operating with decreased service and mobile clinics and counselling services being cancelled (42). There is a risk that the exodus of women from India's workforce may become permanent, reversing not only gender equality gains but also GDP gains unless policies and initiatives intentionally target and invest in women. There is a lack of stable, non-discretionary budget commitments to enable investments in additional infrastructure for emergency services and shelters during the pandemic and policies to allow survivors to move on with their lives in safety, such as housing and gender-sensitive social protection programmes. Non-governmental organisations (NGOs) play an essential role in delivering GBV services, but they should be reinforced with government provision for equitable access (43).

## Various strategies adopted by the public and private sectors in India to prevent GBV in times of COVID-19 in India

While focusing on humanitarian programming, one should concentrate on GBV risk mitigation measures, including training on handling GBV cases to make appropriate and safe referrals. Holistically integrating GBV services (screening, counselling, and referral) into existing maternal and child health services can strengthen the health system's response to GBV. Those providing first-line support must also have access to psychological support. The pandemic highlighted the need to ensure last-mile delivery of essential services and create a robust information-sharing network (5, 44–46). Women and girls need to be recognized as change agents to strengthen programs focused on GBV prevention. Plans should also include measures to strengthen services such as one-stop centers, hotlines, and reporting mechanisms that offer psycho-social support. There is an urgent need to prioritize the coordination of programs and policies across sectors such as health, legal, social welfare, and education to address the composite needs of victims/survivors of GBV (47) By engaging women at the center of all policy-making, one can prioritize interventions that look at increasing economic opportunities for women and address the financial barriers they face (48). The pandemic highlighted the digital-gender divide as only 25% of India's total adult female population owned a smartphone, as opposed to 41% of men in 2020 (49). Digital inclusion could be a key to preventing GBV by creating a robust system of telephonic support counselling and therapeutic interventions that can help support GBV survivors without alerting the perpetrators (50). The pandemic highlighted the need to work with women and civil society organizations (CSOs) to amplify their voices and make them heard. Rapid response teams need to have close relationships with CSOs working on GBV (51). Models like multi-year flexible funding can be explored to ensure support to survivors guided by feminist movements and organizations working, especially on ending GBV (52).

Due to the complexity of GBV programming and health facilities prioritizing COVID-19 response, the stakeholders involved in GBV mitigation and treatment adopted ***some key approaches/strategies in three domains- private, public, and cyberspace*.**

In the **private sphere**, the organizations have reiterated that the pandemic had further exacerbated intimate partner violence for women who earlier sought the support of friends and family for protection from their partners. With the health and support services being scaled back, the chances of receiving support from the health sector grew rare (53). In these trying times, NGOs and CSOs strengthened peer networks *via* WhatsApp, Whisper Circles, etc., towards improving health-seeking behavior. “Whisper circles" became an effective tool to identify violence cases and helped connect the survivors with counsellors. Organizations like Aangan India (54) protecting young girls from early marriages reinforced their early warning system so that information was restricted to violence. They received details on girls at risk for child marriage, which was later passed on to the helplines or facilities for action (55). The organizations made efforts to reach survivors who had been refrained from support due to being in abusive relationships. Targeted efforts were also made to understand the impact of COVID-19 on mental health and steps towards improving access to online mental health services.

II. In the **public sphere,** the Ministry of Women and Child Development ensured that One-Stop centers providing legal and psychosocial support to survivors of GBV were integrated with local medical teams, police, shelter home, working women hostels, and mental health hospitals. The Ministry also urged non-governmental organizations to undertake individual calls with at least ten women a day so that they knew they were not alone (56). Organizations worked towards amplifying the usage of state, and national-level women/child-friendly helplines. The National Commission for Women launched a unique Whatsapp number to report domestic violence cases during the lockdown, urging grassroots organizations to amplify the number on the ground (57). The helplines offering psychosocial support and legal services proved catalytic towards supporting survivors of GBV. Actionaid Association set up COVID-19 emergency helpdesks across 90 districts and supported some helplines with assistance from medical professionals providing telemedical consultation and psycho-social counselling (48). It became apparent that during the lockdown, various schemes initiated by the Government of India pointed toward the need for strategies that follow a discreet method of reporting (58). State government's initiatives and campaigns picked up across the country to signal intolerance for GBV. These were the “Suppress corona, not your voice” campaign of Uttar Pradesh, the Police's Phone-Up program of Odisha, and the opening of a women's tele-counselling facility by the Kerala State Commission (59).

III. A comparative analysis of data from NFHS-4 and NFHS 5 has demonstrated that there has been a small increase in women's access to mobile phones over the last five years. The figure has gone up from 45.9 per cent to 54 per cent between the two surveys while **digital adoption** took a quantum leap in India during the pandemic, this also led to increased cyber-violence, and online and ICT facilitated violence against women and girls.

Digital Intelligence Report 2021 by International Center for Research on Women (ICRW) reported that the size of the conversation around online violence on Twitter nearly tripled from pre- to during COVID. Urban cities -Bengaluru, Karnataka, Kolkata, Mumbai, and New Delhi, reported exponential growth in interest towards doxing, online stalking, gender trolling, image-based abuse, online sexual harassment, etc. during COVID (60). Some organizations worked proactively to leverage the support of social media influencers to spread awareness about GBV, voice up, and seek assistance. They worked towards organizing dedicated social media campaigns to combat online and offline GBV. Understanding the gravity of the situation, the experts mentioned that rallying the support of influential people helped create momentum against GBV, and the information could be disseminated to a larger audience giving the issue more visibility. Also, the use of textual and visual identifiers helped the campaigns on social media get extensive reach to push for effective discourse and raise awareness for women in lockdown facing GBV. Recognizing the importance of offline activities due to the prevailing digital gender divide, several organizations also tried safeguarding adolescents through peer safety networks and formulation “Girl Power Groups", which remained at the frontline of stopping child marriage in India. One of the fundamental reasons for the digital gender divide is the intersection between economic adversity and gender bias. Due to the family's scarce resources, the education of the male child is prioritized as opposed to the female, who is expected to contribute to domestic chores (49). Social media has become a powerful help tool for the dissemination of information. The use of social media to spread the news on violence against women and assistance with available services was helpful in times of the pandemic wherein restrictions do not allow women to move about to seek help (61). During COVID, Akshara Centre, a women's rights organization, Tata Institute of Social Sciences (RCI-VAW), and the Department of Women and Child (Government of Maharashtra) started an app called “Stand-up against violence”. This app mapped out the mobile numbers of State and non- State agencies, service providers, and women's movement groups responding to violence against women across Maharashtra at the subdistrict level, making it easier for women survivors and supporters to seek assistance (51). Some local organizations thought of interesting technological solutions as an effective strategy to mitigate GBV. Feminist Approach to Technology initiated “ Corona Nahi, Karuna!" (Spreading Compassion, not Corona) where they connected young girls to their ongoing programs online. A human chain of campaigners was formed to check on vulnerable girls regularly (62). Angan Trust equipped an already trained and mobilized cadre of community child protection volunteers with smartphones and developed easy to use digital tools so they could be responsive despite the lockdown (63).

## Recommendations

A. Government and policy makers.
  • There is requirement to change systems and create mechanisms that promote gender-responsive structures and inform decision-makers to ensure linkages across government ministries/departments for a systematic change towards preventing GBV.
  • Adequate public resources must be allocated for GBV prevention, risk mitigation, and response.
  • Public planning, budgeting, and financial systems need to integrate gender equality principles and gender analysis (63).
  • Development of country/state strategic plans for preparedness and responses need to be adequately guided by gender analysis.
  • Urgent need to prioritize the coordination of programs and policies across sectors such as health, legal, social welfare, and education to address the composite needs of victims/survivors of GBV.

Potential measurable indicators include:

  
  • Proportion of existing national legislation to promote gender equality, women and child's rights protection.
  • Number of active public institutions working towards combating GBV.
  • Number of public awareness campaigns to raise awareness on GBV, legal rights of women and challenge harmful social norms.
  • Number of public institutions that maintain gender disaggregated data systems.
  • Increased number of females in decision-making positions attained through policy intervention.
  • Number of policies and guidelines placed and effectively implemented to ensure gender concerns are addressed in rescue, relief, rehabilitation, and reconstruction stages of natural disasters or humanitarian emergencies.

Amount of allocations made to complement Multi Sectoral National Action Plans to address sexual and gender-based violence.

B. Donors/Funding Organizations/Philanthropic Foundations.
  • To increase the number of innovative investment opportunities for private or blended capital (a mix of grants, equity investments, and bank loans) related to shifting harmful GBV outcomes.
  • To promote localization of humanitarian assistance, and support women's and girls' leadership and participation around all areas of program/policy design and implementation.
  • Removing bottlenecks to ensure funding can reach grassroots organizations working closely with vulnerable communities.
  • Exploring innovative financing pilots to support localized GBV programming.

Potential measurable indicators:

  
  • Proportion of funds allocated by international funding organizations investing in prevention, risk mitigation and response towards GBV.
  • Proportion of innovative models to reduce GBV scaled to impact at local level.
  • Amount of allocations made to complement multisectoral national /state action plans to address gender-based violence.
  • Number of active platforms created which amplify voices of women and adolescent girls.
  • Number of new funding grant opportunities generated to fund gender centric and sustainable solutions.
C. Civil Society/Humanitarian Organizations.
  • Humanitarian and development organisations should aim at strengthening context-specific partnerships to carry out joined-up regional, country or area-based assessment, planning and programming on GBV.
  • Awareness-raising materials and approaches should be context-specific, and inclusive in nature depending on local vulnerabilities.
  • Need to develop innovative approaches for research within in the context of COVID-19 in order to better understand the dimensions of GBV in affected settings.
  • Usage of data disaggregated by sex, age, and disability, in order to better understand the differential experiences of affected individuals and communities, and to guide gender-informed action in the short, medium and long-term.

Potential measurable indicators include:

  
  • Number of gender discriminatory laws, policies and procedures that are amended, enacted, and/or implemented due to advocacy efforts by civil society organizations.
  • Proportion of community level prevention and response mechanism which are effective and active on the ground.
  • Number of intersectional analysis undertaken to provide a more comprehensive picture necessary to trigger policy and programmatic shifts.

Percentage of data sets focussing on how the health and socioeconomic effects of COVID-19 have exacerbating gender-based bias, discrimination, mistreatment, abuse, violence, norms, and the agency of women and girls.

Generatingsex-disaggregated data on impacts of COVID-19 on migrants, covering indicators such as COVID-19 knowledge and behavior, a and social protection or safety nets.

  
  • Sexual and gender-based violence report rate, based on the number of incidents of sexual and gender-based violence in a population during a designated time period (month, year etc), expressed as a number of incidents per 10,000 persons during that time period.
  • Percentage of civil society organizations working towards ensuring accountability of government to respond effectively to gender-based violence.
D. Service Providers
  • Holistically integrating GBV services (screening, counselling, and referral) into existing maternal and child health services strengthens the health system's response to GBV.
  • Providing first-line support must also have access to psychological support.
  • Provision of adequate training for health workers to handle disclosures of GBV.
  • Identify support systems that are important for GBV survivors to seek help and stop incidents of violence.
  • Ensuring last-mile delivery of essential services and creating a robust information-sharing network on GBV.
  • Potential measurable indicators include:
  • Proportion of public and private health service delivery points providing empowerment counselling and psycho social support for GBV survivors.
  • Number of frontline workers/ community service providers trained on gender sensitive delivery standards.
  • Percentage of clients identified for GBV care who receive post- GBV clinical case based on minimum package.
  • Percentage of people identified for GBV care who receive first line response (LIVES) according to WHO guidelines.
  • Number of healthcare professionals capacitated on gender competencies /gender sensitive quality care.
  • Percentage of providers that demonstrate survivor centred care in GBV screening and first-line response.
  • Percentage of facilitates that meet the minimum requirements for screening for GBV per WHO guidelines.

## Conclusion

The COVID-19 pandemic uncovered the existing gaps in gender programming across the world. Fragmented across sectors, efforts to end violence are sometimes ineffective as they fail to address the intersectional forces that play into GBV. The pandemic also highlighted the need to strengthen accountability and build capacities of local women's organizations to ensure women's voices are heard and amplified. Recognizing the scale and dimensions of GBV within the context of COVID-19, it is imperative that impact and effectiveness of existing and adapted GBV prevention, response, and risk mitigation approaches is reassessed at local, state and national level in India.
